# Novel CD200 homologues iSEC1 and iSEC2 are gastrointestinal secretory cell-specific ligands of inhibitory receptor CD200R

**DOI:** 10.1038/srep36457

**Published:** 2016-11-07

**Authors:** Toshiyuki Kojima, Kiichiro Tsuchiya, Shinji Ikemizu, Soichiro Yoshikawa, Yoshinori Yamanishi, Mamoru Watanabe, Hajime Karasuyama

**Affiliations:** 1 Department of Immune Regulation, Tokyo Medical and Dental University, 1-5-45 Yushima, Bunkyo-ku, Tokyo, 113-8519, Japan; 2 Department of Gastroenterology and Hepatology, Tokyo Medical and Dental University, Graduate School of Medical and Dental Sciences. Tokyo 113-8519, Japan; 3 Division of Structural Biology, Graduate School of Pharmaceutical Sciences, Kumamoto University. Kumamoto 862-0973, Japan

## Abstract

CD200R is an inhibitory receptor expressed on myeloid cells and some lymphoid cells, and plays important roles in negatively regulating immune responses. CD200 is the only known ligand of CD200R and broadly distributed in a variety of cell types. Here we identified novel CD200 homologues, designated iSEC1 and iSEC2, that are expressed exclusively by secretory cell lineages in the gastrointestinal epithelium while authentic CD200 is expressed by none of epithelial cells including secretory cells. Both iSEC1 and iSEC2 could bind to CD200R but not other members of the CD200R family. Notably, CD200R expression was confined to intraepithelial lymphocytes (IELs) among cells in the gastrointestinal epithelium. Binding of iSEC1 to CD200R on IELs resulted in the suppression of cytokine production and cytolytic activity by activated IELs. Thus, iSEC1 is a previously unappreciated CD200R ligand with restricted expression in gastrointestinal secretory cells and may negatively regulate mucosal immune responses.

Paired receptors are closely related membrane proteins that share similar extracellular regions but have distinct transmembrane and cytoplasmic regions, and regulate immune responses by transducing opposing signals, either inhibitory or activating, upon ligand binding^1^,^2^. The CD200R family is one of paired receptor families, and consists of five members in mice, one inhibitory receptor (CD200R) and four CD200R-like receptors with activating activity (termed either CD200R2-5 or CD200RLa-e)^3^,^4^,^5^,^6^,^7^,^8^,^9^,^10^. CD200 is the only known ligand of the CD200R family, and has been shown to bind to CD200R but no other members^4^,^7^,^11^. CD200 is broadly distributed in a variety of cell types including non-hematopoietic cells whereas CD200R is primarily expressed in myeloid and lymphoid cells^6^,^12^. Both CD200 and CD200R contain two extracellular immunoglobulin superfamily (IgSF) domains and a single-pass transmembrane region, and interact with each other through their N-terminal IgSF domains^13^. The CD200R intracellular domain lacks the typical immunoreceptor tyrosine-based inhibitory motif (ITIM) present in most immune inhibitory receptors, but it contains three tyrosine residues that can be phosphorylated and contribute to inhibitory signaling^14^. Recent studies, including analyses with CD200R- or CD200-deficient mice, have demonstrated that the CD200-CD200R interaction plays important roles in negatively regulating immune responses, and attenuate autoimmune diseases, excessive inflammatory responses against pathogens, or anti-tumor immunity^9^,^15^,^16^,^17^,^18^,^19^,^20^.

Functional roles of CD200R-like receptors have been ill-defined compared to those of CD200R. Among four members, CD200R3 (CD200RLb) is unique in terms of structure and expression. It exists as a disulfide-linked homodimer unlike other members, and its expression is restricted to mast cells and basophils^7^. Cross-linking of CD200R3 activates these cells through an adaptor molecule DAP12 to degranulate and produce cytokines such as IL-4^7^ while CD200R signal inhibits them^21^,^22^. In spite of intensive investigation, no apparent ligands have been identified for CD200R-like activating receptors.

The surface of mucosa and skin represents a first line of defense against invading pathogens. The gastrointestinal tract is constantly and heavily loaded with non-self substances, including food antigens, commensal bacteria, and pathogenic organisms. From the stomach to the rectum, the mucosa consists of a single layer of columnar epithelial cells, organizing into crypts that invaginate into the underlying mesenchyme, and villi that project into the intestinal lumen. Intestinal stem cells reside near the bottom of crypts, and differentiate into distinct types of epithelial cells, including absorptive enterocytes and multiple secretory cells (goblet cells, enteroendocrine cells, and Paneth cells)^23^,^24^. Goblet cells and enteroendocrine cells secrete mucus and a variety of hormones, respectively, and occur both in villi and crypts. Paneth cells at the bottom of crypts secrete bactericidal products such as lysozyme and defensins, and also provide the stem cell niche. Intraepithelial lymphocytes (IELs) are a unique subset of intestinal T cells, and located in the epithelial layer as single cells in tight association with intestinal epithelial cells, with about one T cell for every four to nine epithelial cells in the small intestine^25^,^26^,^27^. In contrast to conventional T cells, IELs are enriched in T cell receptor γδ- and CD8αα-expressing cells, and play important roles through their intimate interaction with intestinal epithelial cells in the maintenance of mucosal homeostasis by actively or negatively regulating mucosal and acquired immunity.

In the present study, we have identified novel CD200 homologues, designated iSEC1 and iSEC2, that showed the ability of binding to CD200R but not to CD200R-like receptors. Intriguingly, the expression of iSEC1 was confined to secretory cell lineages in gastrointestinal epithelial cells. Only IELs expressed CD200R among cells in the intestinal epithelium, and none of intestinal epithelial cells expressed CD200, suggesting possible interaction between CD200R on IELs and iSEC1 on secretory cells in the intestinal epithelium. Binding of iSEC1 to CD200R on IELs indeed attenuated the cytokine production and cytolytic activity of activated IELs, demonstrating that iSEC1 is a functional ligand of CD200R, and may negatively regulate the function of IELs.

## Results

### Identification of novel CD200 homologues, designated iSEC1 and iSEC2

**Figure 1:**
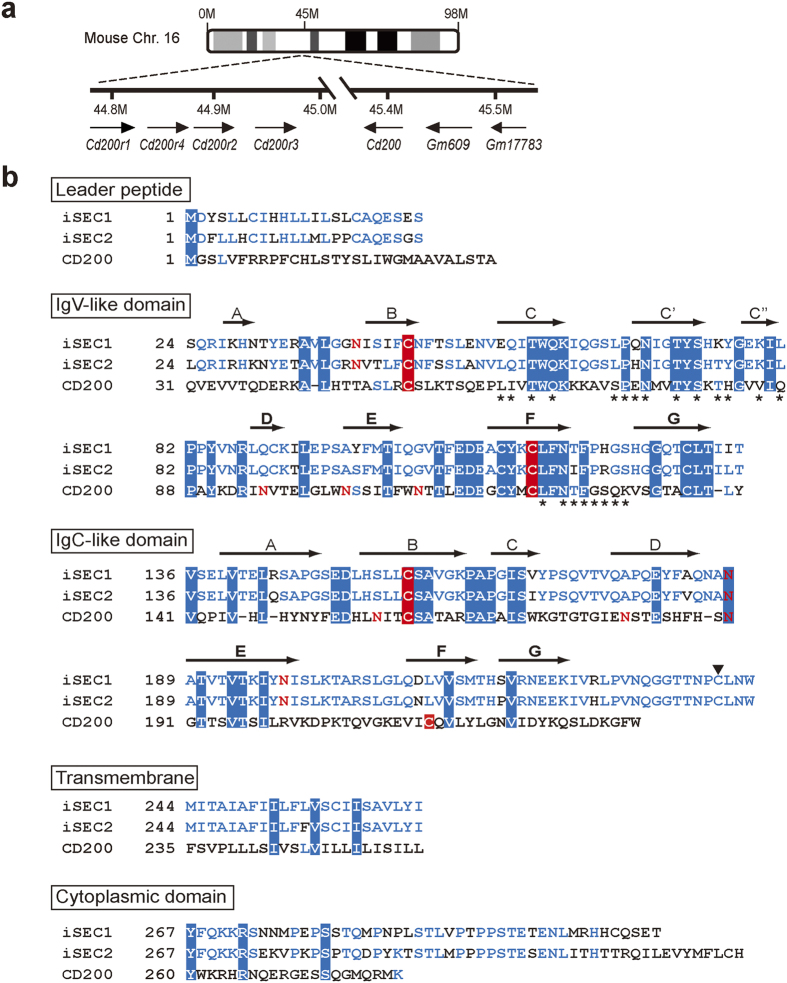
Identification of two genes encoding CD200 homologues, designated iSEC1 and iSEC2. (**a**) The chromosomal localization of the *Gm609* and *Gm17783* genes next to the *Cd200* gene. (**b**) Amino acid sequences of iSEC1, iSEC2, and CD200 are aligned according to the predicted secondary structure and domain organization based on the known CD200 structure. Residues shared by all three proteins are highlighted in blue, and conserved cysteine residues for IgSF-domain formation are highlighted in red. Residues identical between only two proteins are indicated by blue letters. Asterisks denote residues at the CD200/CD200R interface^13^. A cysteine residue involved in the disulfide-linked homodimer formation of iSEC1 and iSEC2 is indicated by an arrowhead. Potential N-glycosylation sites are denoted by red letters.

With the aim of identifying a ligand(s) of CD200R-like receptors, we first performed an *in silico* screening of CD200 homologues by using NCBI BLAST. Amino acid sequences deduced from nucleotide sequences of three clones (Gm609, Gm17783, and RIKEN9130202L22) showed the highest score when subjected to pairwise alignment analysis with the CD200 sequence. Among three, Gm609 and RIKEN9130202L22 clones showed overlapping nucleotide sequences, most likely representing the same gene while Gm17783 displayed a nucleotide sequence highly homologous to but distinct from that of other two clones, suggesting the existence of two closely related genes. Searching NCBI database of the mouse genome revealed that the Gm609 and Gm17783 genes are adjacent to each other on chromosome 16, and located next to the CD200 gene (Fig. 1a), strongly suggesting that these two genes may have been generated through gene duplication, and could encode CD200 homologues. Because the nucleotide sequence of the Gm609/RIKEN9130202L22 gene available in the database seemed incomplete when compared to that of the Gm17783 gene, we cloned cDNAs corresponding to the Gm609/RIKEN9130202L22 gene as well as those of the Gm17783 gene, and determined their nucleotide sequences (accession no. AB853320 and AB853321, respectively).

As demonstrated later, proteins encoded by Gm609/RIKEN9130202L22 and Gm17783 genes show a unique expression profile, and therefore we designated them iSEC1 (intestinal secretory cell-expressed 1) and iSEC2, respectively. iSEC1 and iSEC2 proteins predicted from the nucleotide sequences of cloned cDNAs consist of 312 and 320 residues, respectively, including a 23 a.a.-long leader peptide in both, showing high identity to each other (83.3%) (Fig. 1b). When compared as a whole, iSEC1 and iSEC2 show relatively low identity to CD200 (23.7% and 24.5%, respectively). Nevertheless, according to the Conserved Domain Database, IgV-like domains of iSEC1 and iSEC2 correspond to the Ig1_MRC-OX-2_like domain (accession no. cd05846) of CD200. They have amino acid sequences conserved in CD200 molecules derived from different animal species and viral homologues, including in the regions corresponding to the C, C’, C”, and F strands of CD200 that are involved in binding to CD200R (Fig. 1b and Supplementary Fig. 1). When compared to CD200, both iSEC1 and iSEC2 lack one of canonical cysteine residues necessary for an intramolecular disulfide bond in the IgSF-C2 domain, while two canonical cysteine residues in the IgSF-V domain are conserved. Importantly, both iSEC1 and iSEC2 carry two extra cysteine residues in F and G strands that is a particular feature of CD200 (indicated by red arrow heads in Supplementary Fig. 1). Taken together, iSEC1 and iSEC2 appear to be previously-unappreciated homologues of CD200. Searching the NCBI database identified iSEC1 orthologues in many other animal species that express CD200 orthologues as well (Supplementary Fig. 2).

### iSEC1 and iSEC2 bind to CD200R but do not any of CD200R-like receptors

**Figure 2:**
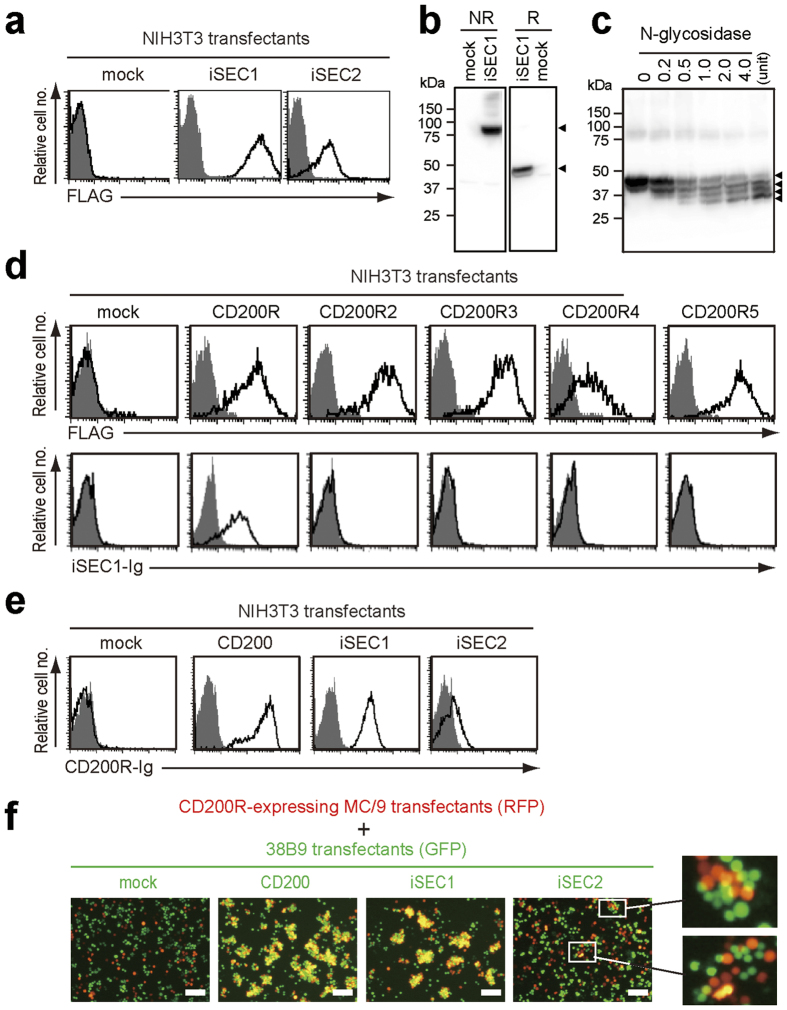
iSEC1 and iSEC2 proteins exist as disulfide-linked homodimers and bind to CD200R but not CD200R-like receptors. (**a–c**) NIH3T3 cells were infected with retroviral vectors encoding FLAG-tagged iSEC1 or iSEC2, or mock. In (**a**) transfectants were subjected to flow cytometric analysis for surface expression of iSEC1 or iSEC2 detected with anti-FLAG. Shaded histograms indicate control staining with isotype-matched control Ab. In (**b**) cell lysates of transfectants were subjected to SDS-PAGE under non-reducing (NR) or reducing (R) conditions, and then immunoblot analysis to detect FLAG-tagged iSEC1, as indicated by arrowheads. In (**c**) cell lysates of iSEC1 transfectants were treated with indicated units of N-glycosidase, and subjected to SDS-PAGE under reducing conditions, and then immunoblot analysis as in b. (**d**) NIH3T3 cells infected with retroviral vectors encoding FLAG-tagged CD200R family proteins, or mock were stained with anti-FLAG (open histograms) or isotype-matched control Ab (shaded histogram) to demonstrate surface expression of FLAG-tagged proteins (upper panels). In lower panels, the transfectants were incubated with iSEC1-IgFc fusion proteins (open histograms) or control mock IgFc (shaded histograms), followed by detection of bound fusion proteins with anti-IgG1. (**e**) NIH3T3 transfectants expressing mock control, CD200, iSEC1 or iSEC2 were incubated with CD200R-IgFc (open histograms) or control FLAG-IgFc (shaded histograms) fusion proteins, followed by detection of bound fusion proteins with anti-IgG1. (**f**) MC/9 transfectants expressing RFP and CD200R (Supplementary Fig. 4) were co-cultured for 24 h with 38B9 transfectants expressing GFP together with CD200, iSEC1, or iSEC2 (Supplementary Fig. 4), and the formation of red (RFP) and green (GFP) cell aggregates was examined under fluorescence microscope. Bar = 100 μm. Data shown in (**a–f**) are representative of at least three independent experiments.

For biochemical characterization of iSEC1 and iSEC2 proteins, a fibroblast cell line NIH3T3 was transfected with retroviral vectors to express FLAG-tagged iSEC1 or iSEC2 (Fig. 2). As expected from the presence of a transmembrane region, both proteins were detected on the cell surface of transfectants (Fig. 2a). The surface expression of iSEC2 was reproducibly much lower than that of iSEC1 (Fig. 2a), even though the fluorescence intensity of co-expressed GFP was comparable in iSEC1- and iSEC2-transfectants (data not shown). Western blot analysis revealed that both FLAG-tagged iSEC1 and iSEC2 proteins had apparent molecular masses of 82 and 43 kDa under non-reducing and reducing conditions, respectively (Fig. 2b), indicating that they formed disulfide-linked homodimers. The amount of iSEC2 proteins estimated from the band intensity was approximately 50 times less than that of iSEC1 at a per cell level (data not shown). When differentially-tagged iSEC1 and iSEC2 were co-expressed in NIH3T3 cells, heterodimers of iSEC1 and iSEC2 were detected as a minor fraction while vast majority of products were iSEC1 homodimers (data not shown). Treatment with N-glycosidase reduced an apparent molecular mass of iSEC1 proteins, resulting in the appearance of three smaller bands (41, 38, 35 kDa) under reducing conditions (Fig. 2c), in accordance with the presence of three potential N-linked glycosylation sites (Fig. 1b).

Only CD200R3 among the CD200R family exists as a disulfide-linked homodimer^7^. Therefore, we assumed that homodimeric forms of iSEC1 and iSEC2 could be ligands of CD200R3. However, a fusion protein of the extracellular region of iSEC1 and the Fc region of IgG1 showed no detectable binding to any of CD200R-like receptors, including CD200R3, expressed on NIH3T3 cells (Fig. 2d). Intriguingly, the iSEC1-IgFc fusion protein could bind to CD200R on NIH3T3 transfectants (Fig. 2d). In accordance with this, CD200R-IgFc fusion proteins showed clear binding to iSEC1 as well as CD200 expressed on NIH3T3 cells (Fig. 2e). Importantly, pre-incubation of CD200R-expressing NIH3T3 transfectants with CD200-IgFc proteins inhibited the binding of not only CD200 but also iSEC1 to CD200R expressed on the transfectants (Supplementary Fig. 3), suggesting that iSEC1 and CD200 bind to the same or very similar site of CD200R. Although we could not get enough amounts of iSEC2-IgFc fusion proteins for binding analysis, due to its poor expression in transfectants, CD200R-IgFc fusion proteins showed significant binding to iSEC2 expressed on NIH3T3 cells (Fig. 2e). By contrast, none of CD200R2-IgFc, CD200R3-IgFc, CD200R4-IgFc or CD200R5-IgFc fusion proteins showed any detectable binding to iSEC1 or iSEC2 on NIH3T3 cells (data not shown). These results suggested that iSEC1 and iSEC2 are novel ligands of CD200R rather than CD200R-like receptors. To examine the interaction between membrane-bound forms of CD200R and iSEC1/2, non-adherent cells co-expressing CD200R and RFP were co-cultured with another cells expressing either mock, CD200, iSEC1 or iSEC2 together with GFP (Fig. 2f and Supplementary Fig. 4). iSEC1-expressing cells formed large cell aggregates with CD200R-expressing cells as did CD200-expressing cells. Much smaller but significant aggregates were formed by iSEC2- and CD200R-expressing cells. These results strongly suggested that both iSEC1 and iSEC2 could be involved in cell-cell interaction through CD200R.

### iSEC1 proteins are selectively expressed by secretory cell lineages in the gastrointestinal tract

**Figure 3:**
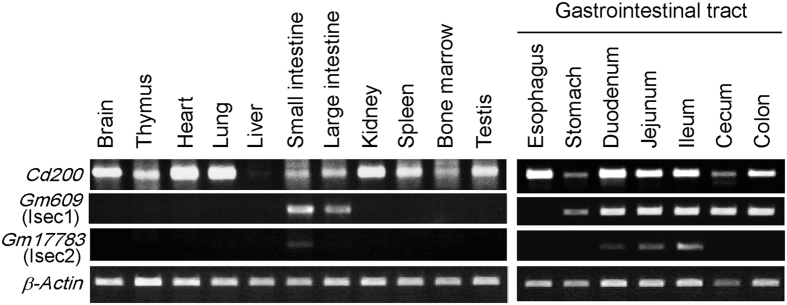
Transcription of *Gm609* and *Gm17783* genes is confined to the gastrointestinal tract. The expression of *Cd200*, *Gm609* and *Gm17783* mRNAs in indicated tissues was analyzed with RT-PCR in that PCR templates were adjusted based on the expression of *Actb* mRNAs. Compiled photographs of gel trips containing bands of PCR products corresponding to indicated mRNAs are shown as representative of three independent experiments.

The EST database suggested that the transcription of the Gm609/RIKEN9130202L22 gene encoding iSEC1 is confined to the intestine (UniGene, Mm.451852/EST profile), in contrast to rather ubiquitous expression of the CD200 gene (UniGene, Mm.245851/EST profile). In accordance with this, Gm609/RIKEN9130202L22 mRNAs were detected in the stomach and the small and large intestine but not the esophagus or other tissues examined (Fig. 3), indicating that the Gm609/RIKEN9130202L22 gene is selectively expressed in the digestive tract lined with columnar epithelium. The expression of the Gm17783 gene encoding iSEC2 was even more selective, and restricted to the small intestine (duodenum, jejunum and ileum) (Fig. 3).

**Figure 4:**
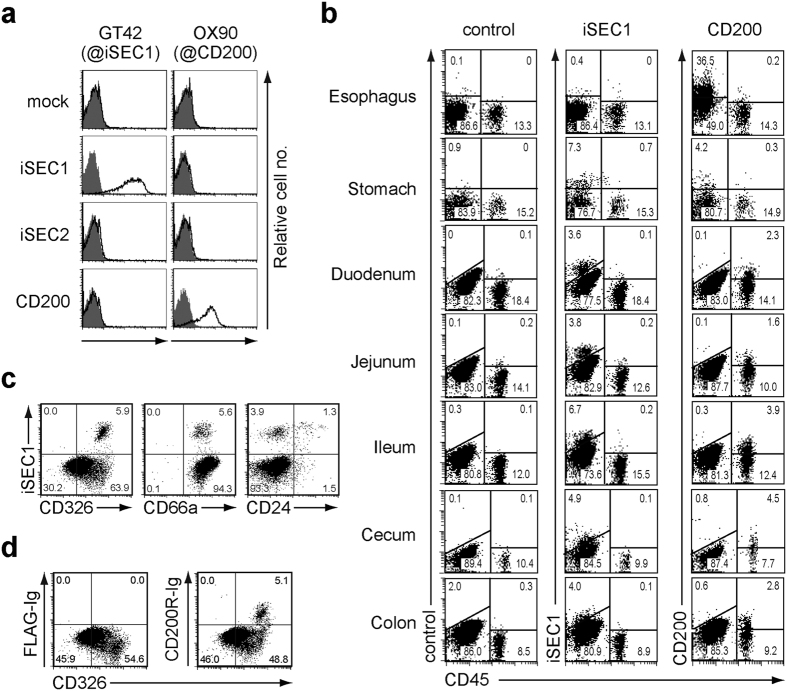
iSEC1 is expressed by a small fraction of CD45^−^ epithelial cells in the gastrointestinal tract. (**a**) NIH3T3 transfectants expressing mock control, iSEC1, iSEC2, or CD200 were stained with GT42 or anti-CD200 (OX90) mAb (open histograms), or isotype matched control antibody (shaded histograms). (**b**) Cells isolated from indicated gastrointestinal tissues were stained with anti-CD45 in combination with anti-iSEC1 (GT42), anti-CD200 (OX90) or isotype-matched control. (**c**) CD45^−^ cells isolated from the jejunum epithelium were analyzed for the expression of iSEC1 in combination with CD326, CD66a or CD24. (**d**) Cells isolated from the jejunum epithelium were incubated with CD200R-IgFc or control FLAG-IgFc fusion proteins, followed by detection of bound fusion proteins with anti-IgG1 in combination with CD326 and CD45 staining. CD45^−^ cells were gated for analysis shown here. Data shown in (**a–d**) are representative of three independent experiments.

With the aim of clarifying the nature of cells expressing iSEC1 and iSEC2 in the gastrointestinal tract, we sought to establish monoclonal antibodies specific to them by repeatedly immunizing rats with iSEC1-expressing NIH3T3 transfectants. One mAb, GT42, recognized iSEC1 but not iSEC2 or CD200 expressed on NIH3T3 transfectants, while an anti-CD200 mAb (OX90) reacted with CD200 but not iSEC1 or iSEC2 (Fig. 4a). Flow cytometric analysis with GT42 revealed that the iSEC1 expression was confined to a small fraction (4–9%) of CD45^−^ non-hematopoietic cells in the stomach, duodenum, jejunum, ileum, cecum, and colon, and that the highest level of iSEC1 expression was detected in the small intestine among them (Fig. 4b). In sharp contrast, CD200 expression was barely detected on CD45^−^ cells in the intestine even though it was detected on some of CD45^+^ hematopoietic cells in the intestine and CD45^−^ cells in the esophagus and stomach (Fig. 4b). No apparent expression of iSEC1 was detected in the esophagus or tissues other than the gastrointestinal tract (Fig. 4b and Supplementary Fig. 5). Thus, two different types of CD200R ligands appear to be differentially expressed among gastrointestinal cells, and the ligand expression on epithelial cells shifts from CD200 to iSEC1 along the way from the esophagus to the intestine.

**Figure 5:**
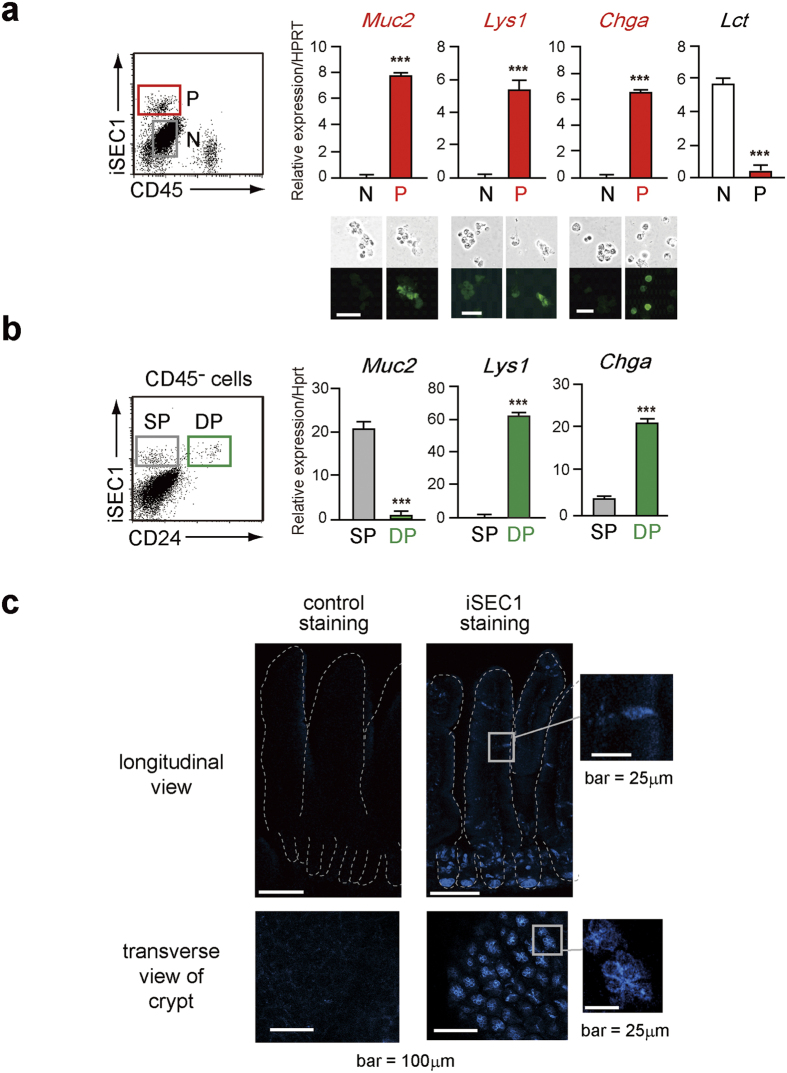
iSEC1 is selectively expressed by secretory cell lineages among intestinal epithelial cells. (**a**) Cells isolated from the jejunum epithelium were stained with anti-CD45 and anti-iSEC1, and CD45^−^iSEC1^+^ (P) and CD45^−^iSEC1^−^ (N) cells were separately sorted, and subjected to quantitative RT-PCR analysis for the expression of indicated mRNAs (mean ± SEM, n = 3) and to cytoplasmic staining for indicated proteins. (**b**) CD24^−^iSEC1^+^ (SP) and CD24^+^iSEC1^+^ (DP) fractions of CD45^−^ cells in the jejunum epithelium were separately sorted, and subjected to the RT-PCR analysis as shown in **a** (mean ± SEM, n = 3). (**c**) Mice were treated with intraperitoneal injection of fluorescent-labeled anti-iSEC1 or control antibody, and the jejunum was isolated and analyzed for iSEC1 expression under confocal fluorescence microscope. Upper panels show longitudinal views of villi and crypts while lower panels display transverse views of crypts. iSEC1-positive cells were stained in blue. Data shown in **a–c** are representative of three independent experiments. ***p < 0.001.

All iSEC1^+^ cells in the jejunum expressed epithelial markers CD326 and CD66a. Importantly, 5~10% of CD45^−^ cells in the jejunum showed the binding of CD200R-IgFc fusion proteins, and they displayed the high level of CD326 (Fig. 4d) as did iSEC1^+^ cells (Fig. 4c). These results suggested that a small fraction of intestinal epithelial cells express iSEC1 that can bind to CD200R. While no specific surface makers are available to clearly distinguish enterocytes, goblet cells, Paneth cells, and enteroendocrine cells among intestinal epithelial cells, it has been shown that CD24 is selectively expressed by Paneth and enteroendocrine cells among them^28^. We found that about one-third of iSEC1^+^ cells displayed CD24, implying that iSEC1^+^ cells include Paneth and/or enteroendocrine cells. This finding, together with the minority of the iSEC1^+^ population among epithelial cells, prompted us to examine the possibility that iSEC1 expression is confined to secretory cell lineages. Indeed, PCR and immunofluorescent analyses revealed that iSEC1^+^ but not iSEC1^−^ cells among CD45^−^ cells in the jejunum epithelium expressed mucin 2, lysozyme and chromogranin A, that are markers of goblet cells, Paneth cells, and enteroendocrine cells^29^, respectively, at both mRNA and protein levels (Fig. 5a). In contrast, lactase, an enterocyte marker, was predominantly expressed in iSEC1^−^ but not iSEC1^+^ cells (Fig. 5a). Among iSEC1^+^ cells, mucin 2 was predominantly expressed by the CD24^−^ population while lysozyme and chromogranin A were by the CD24^+^ population (Fig. 5b) in accordance of CD24 expression restricted to Paneth and enteroendocrine cells among gastrointestinal epithelial cells^28^. Thus, iSEC1 expression appeared to be confined to secretory cell lineages, including goblet, Paneth, and enteroendocrine cells, among gastrointestinal epithelial cells. Indeed, the whole mount staining of the jejunum illustrated that iSEC1^+^ cells were densely packed in the crypt where Paneth cells reside, and were localized as single cells in the epithelium of villi where goblet and enteroendocrine cells exist (Fig. 5c). iSEC1^+^ cells were barely detected in the crypt of the large intestine (data not shown), consistent with the fact that Paneth cells are present in the small but large intestine. The frequency of iSEC1^+^ cells in villi was also much less in the large intestine than in the small intestine (data not shown). Thus, iSEC1 proteins appeared to be expressed selectively by secretory cells, particularly in the small intestine.

### iSEC1 binding to CD200R on IELs attenuates their cytokine production and cytolytic activity

**Figure 6:**
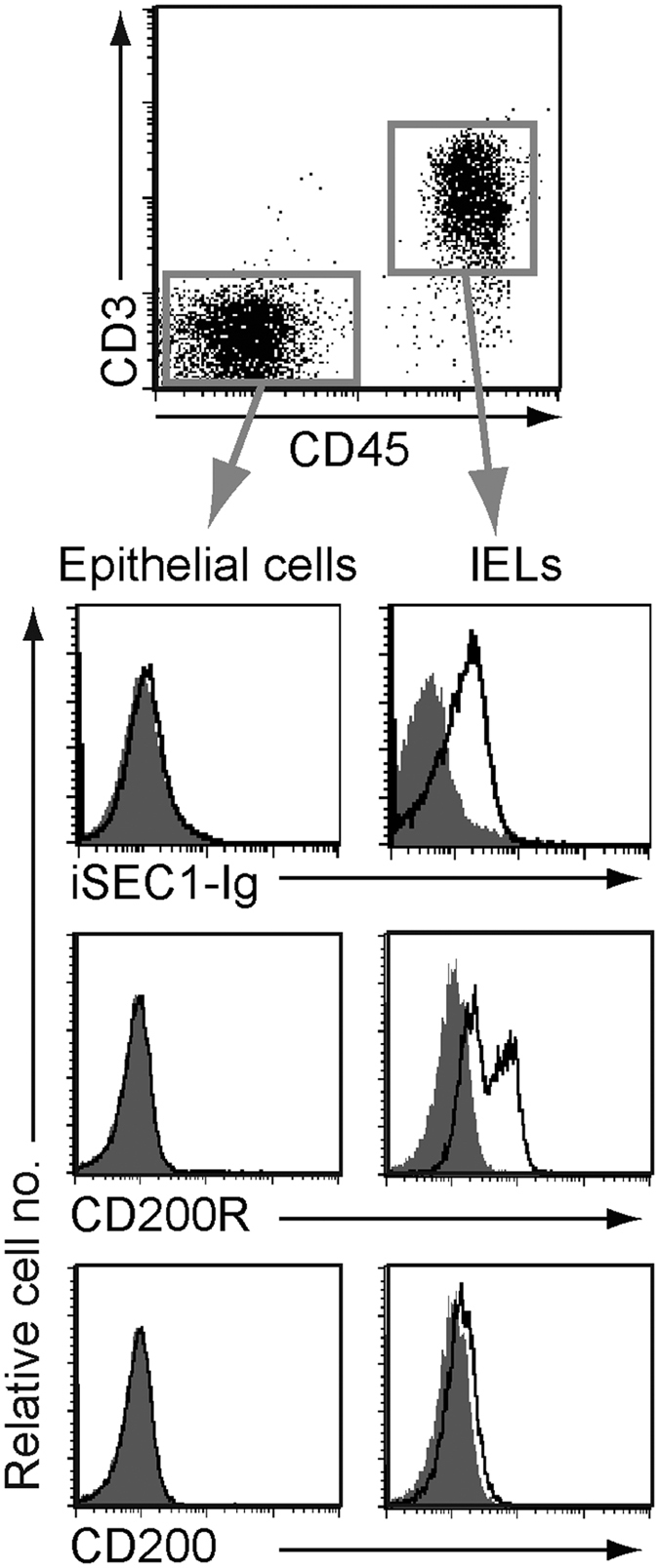
IELs but not epithelial cells constitutively express CD200R and show iSEC1 binding. Cells isolated from collagenase-treated jejunum were stained with iSEC1-IgFc fusion proteins in combination with anti-CD45 and anti-CD3. Shaded histograms show staining with FLAG-IgFc fusion protein as control (upper histogram panels). The lower panels show the expression of CD200R and CD200 in the CD45^+^CD3^+^ T cells and CD45^−^CD3^−^ epithelial cells. Data are representative of three independent experiments.

We next examined what types of cells express a receptor(s) for iSEC1 in the gastrointestinal tract. The CD45^+^CD3^+^ T cell fraction but not the CD45^+^CD3^−^ or CD45^−^ fraction of cells isolated from the jejunum showed the ability to bind iSEC1-IgFc fusion proteins (Fig. 6). In contrast to conventional T cells, this T cell fraction had significant enrichment of T cell receptor γδ-expressing T cells (Supplementary Fig. 6), consistent with the nature of IELs in the intestine. In accordance with the ability to bind iSEC1-IgFc fusion proteins, these IELs including both αβ and γδ T cells, but not CD45^−^ epithelial cells, constitutively expressed CD200R (Fig. 6 and Supplementary Fig. 6) unlike conventional T cells. Notably, the expression of CD200, the authentic ligand of CD200R, was hardly detected in both IELs and epithelial cells in the jejunum epithelium (Fig. 6). Considering the fact that IELs interact intimately with intestinal epithelial cells^30^,^31^, one may assume that the binding of iSEC1 on secretory cells to CD200R on IELs could occur during the cellular interaction.

**Figure 7:**
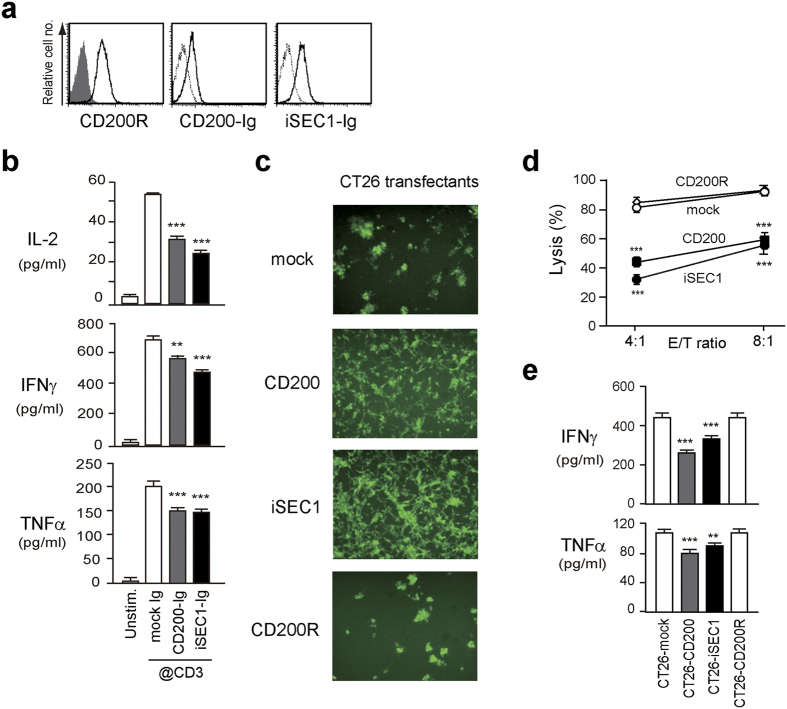
Binding of iSEC1 to CD200R on IELs attenuates their cytokines production and cytolytic activity *in vitro*. (**a**) IL-2-cultured IELs were stained with anti-CD200R, CD200-IgFc, or iSEC-IgFc. A shaded histogram shows staining with isotype-matched control antibody, and dashed histograms show staining with control IgFc. (**b**) IL-2-cultured IELs were starved of IL-2 for 3 h and left unstimulated or stimulated for 24 h with plate-bound anti-CD3 in the presence of plate-bound mock IgFc, CD200-IgFc or iSEC1-IgFc, followed by measurement of indicated cytokines in their culture supernatants (mean ± SEM, n = 3). (**c–e**) IL-2-cultured IELs were incubated at the indicated effector/target (E/T) ratios for 48 hrs with plate-adherent CT-26 transfectants expressing GFP together with mock control, CD200R, CD200, or iSEC1. (**c**) Shows photographs of GFP-expressing CT-26 transfectants (at the 8:1 E/T ratio) taken after the 48 h-cytolysis culture under fluorescence microscope. Cytolysis of CT-26 cells in monolayer culture resulted in detachment and aggregation of cells, as typically observed in mock transfectants (top panel). In (**d**) % lysis of CT-26 transfectants in each group was calculated (mean ± SEM, n = 3). In (**e**) supernatants were harvested from the 48 h-cytolysis culture at the 4:1 E/T ratio, and analyzed for the concentration of indicated cytokines (mean ± SEM, n = 4). Data in (**a**–**e**) are representative of at least three independent experiments. *p < 0.05, **p < 0.01, ***p < 0.001.

To clarify whether iSEC1 indeed plays a role as a functional ligand of CD200R, the effect of iSEC1 binding on the function of IELs was examined. Virtually all of IL-2-cultured IELs employed for this purpose expressed CD200R and could bind iSEC1-IgFc or CD200-IgFc (Fig. 7a). When IELs were stimulated with anti-CD3 mAb, they secreted cytokines including IL-2, IFNγ and TNFα (Fig. 7b). Co-incubation of anti-CD3-stimulated IELs with plate-bound iSEC1-IgFc significantly attenuated the cytokine production, as observed in co-incubation with plate-bound CD200-IgFc, at both protein and mRNA levels (Fig. 7b and Supplementary Fig. 7). We then examined the effect of cellular interaction between IELs and iSEC1-expressing cells to mimic *in vivo* situation. Since it was difficult to maintain and expand *in vitro* secretory cells isolated from the intestine, in contrast to IELs, as surrogates of secretory cells we utilized colon adenocarcinoma cell line CT26 (I-A^d^) infected with the retroviral vector encoding iSEC1 or control proteins. IL-2-stimulated IELs (I-A^b^) showed cytolytic activity against mock-infected CT26 cells (Fig. 7c,d). Importantly, this cytolytic activity of IELs was significantly inhibited when target CT26 cells expressed either iSEC1 or CD200 but not control CD200R (Fig. 7c,d). The production of IFNγ and TNFα from IELs under the same experimental conditions was also attenuated by the expression of iSEC1 or CD200 but not control CD200R on CT26 cells (Fig. 7e). These results suggested that iSEC1 could be a functional ligand of CD200R and negatively regulate the activity of IELs.

## Discussion

CD200 was only the known endogenous ligand of an inhibitory receptor CD200R, while viral homologues of CD200 have been found in several evolutionally diverse viruses, including Herpesviridae and Poxviridae families, most likely as the result of the CD200 gene acquisition from infected host cells^22^,^32^,^33^. In the present study, we have identified two membrane-bound proteins in mice, iSEC1 and iSEC2, as novel CD200R ligands that are expressed exclusively in secretory cell lineages of the intestinal epithelium, including goblet cells, enteroendocrine cells, and Paneth cells, in contrast to much broader expression of CD200. Such distinct expression patterns among multiple ligands are also observed in another inhibitory receptor programmed death 1 (PD-1)^34^. PD-L1 expression is found on a wide range of both hematopoietic and non-hematopoietic cells while PD-L2 expression is much more restricted, and detected on macrophages, dendritic cells and mast cells. Of note, the genes encoding PD-L1 and PD-L2 are located side by side on the same chromosome, as in the case of the genes coding for CD200, iSEC1 and iSEC2, suggesting that multiple ligands in both cases may have been created through diversification of the common ancestral gene during evolution.

We demonstrated in the present study that binding of iSEC1 to CD200R on IELs attenuates cytokine production and cytolytic activity by activated IELs *in vitro*. IELs have often been described as being ‘activated yet resting’ or ‘partially activated’ T cells^25^,^30^,^35^, because they show characteristics of both activated and resting T cells. The immunologically quiescent status of IELs is attributed in part to their expression of CD8αα and inhibitory natural killer cell receptors^30^,^31^. In the present study, we showed that virtually all IELs, unlike conventional T cells, constitutively express an inhibitory receptor CD200R, suggesting another mode of IEL suppression through CD200R. In brain, the interaction of CD200R and CD200 expressed by microglia and neurons, respectively, has been shown to play an important role in maintaining microglia in a resting state^3^,^12^,^15^,^36^,^37^. Disruption of the interaction results in enhanced inflammation in the brain. In the intestine, IELs reside as single cells in the epithelial layer along the crypt-villus axis^25^,^30^,^38^,^39^ and migrate dynamically to make extensive contacts with multiple epithelial cells^25^,^30^,^38^. Therefore, one may assume that CD200R^+^ IELs frequently interact with iSEC1^+^ secretory cells in the intestinal epithelium, and the inhibitory signal through CD200R-iSEC1 interaction may contribute to negative regulation of IELs. Moreover, considering that secretory cells play an important role in innate protection against pathogens in the gut^40^,^41^,^42^, the iSEC1 expression on secretory cells may protect themselves from unwanted attack by activated IELs.

In conclusion, the present study has identified previously unrecognized ligands of CD200R, designated iSEC1 and iSEC2, that are expressed exclusively by secretory cells in the gastrointestinal epithelium. Importantly, IELs constitutively express CD200R, and the interaction of CD200R with iSEC1 dampened the activation of IELs. Thus, iSEC1 appears to be a functional ligand of CD200R, and can negatively regulate the function of IELs. Detailed study on the CD200R-iSEC1 inhibitory axis in the intestinal epithelium may cast new light on the homeostatic regulation of mucosal immunity.

## Methods

### Animals

C57BL/6 and BALB/c mice (7–10 week old), and SD rats (8 weeks old) were purchased from CLEA Japan. All animal studies were approved by the Institutional Animal Care and Use Committee of Tokyo Medical and Dental University, and all experiments were carried out in accordance with approved guidelines.

### Cell lines, cell preparation, and culture

The fibroblast cell line NIH3T3 (ATCC CRL-1658) and the murine colon adenocarcinoma line CT26 (ATCC CRL-2638) were cultured in DMEM (Nacalai Tesque) supplemented with 10% FCS (GIBCO**/**Invitrogen), 100 U/ml penicillin, and 100 μg/ml streptomycin. The pro-B cell line 38B9 was maintained in RPMI1640 (Nacalai Tesque) supplemented with 10% FCS, 100 U/ml penicillin, 100 μg/ml streptomycin, 1 mM sodium pyruvate, 0.1 mM non-essential amino acid, 2 mM L-glutamine, and 5 × 10^−5^ M 2-mercaptoethanol. The mast cell line MC/9 (ATCC CRL-8306) was cultured with supplemented RPMI1640 including 0.3 ng/ml IL-3. Single cell suspensions were prepared by treating indicated organs and tissues with collagenase (Wako Pure Chemical Industries Ltd). Cells in the epithelium of the small intestine were prepared by incubating the small intestine with PBS containing 2 mM EDTA and 1 mM DTT to detach the epithelium, followed by treating epithelial cell aggregates with collagenase. IELs were prepared by incubating the small intestine with HBSS containing 1 mM EDTA and 1 mM DTT, followed by Percoll gradient fractionation or by sorting of cells in the lymphocyte gate with FACSAria (BD Biosciences). For functional assays, IELs were cultured and expanded for 2 or 3 weeks in complete IMDM supplemented with 40 ng/ml IL-2.

### Antibodies

Purified anti-mouse CD3 (145-2C11, low toxin azide free), APC/Cy7-conjugated anti-mouse CD3 (17A2), PE/Cy7-conjugated anti-mouse CD24 (M1/69), PE-conjugated anti-mouse CD45 (30-F11), anti-mouse CD66a (MAb-CC1), anti-mouse CD200R (OX-110), anti-mouse CD200 (OX-90), Alexa488-conjugated anti-mouse CD326 (G8.8) and APC-conjugated anti-mouse IgG1 (A85-1) were purchased from Biolegend. FITC-conjugated anti-rabbit IgG and biotin-conjugated anti-mouse IgG1 (A85-1) were from BD Bioscience. HRP- and biotin-conjugated anti-FLAG (M2) were from Sigma-Aldrich. PE/Cy7-conjugated anti-mouse CD45 (30-F11) was from Tonbo bioscience. Anti-histidine Tag antibody was from GenScript. Anti-mucin2 (Mucin 2; sc-15334) and anti-chromogranin A (Chr-A; sc-13090) were from Santa Cruz Biotechnology, and anti-lysozyme (EC 3.2.1.17) was from DAKO.

### Retroviral vectors and infection

cDNAs encoding iSEC1 and iSEC2 were prepared from intestinal cells by means of RT-PCR using following primers: for *Isec1*, forward 5′-aggaattctttcagttcagctaggaaacac-3′ and reverse 5′-aaggctcgagtcacatacaatcaattatg-3′, and for *Isec2*, forward 5′-aggaattcgtaggaggaaattacattgtccg-3′, and reverse 5′-aaggctcgagctactagccaatgcat-3′ in that underlined sequences recognized by restriction enzymes were inserted for subsequent subcloning into the pBC(KS-II) vector (Stratagene). PCR primers for cDNAs encoding CD200 and CD200R1-5 were previously described^7^. cDNAs of FLAG-tagged products were inserted into the retroviral vector pMX-IRES-GFP. Soluble forms of membrane-bound proteins were prepared by constructing hybrid cDNAs coding for the extracellular part of CD200, CD200R1, or iSEC1, and the Fc region of mouse IgG1 (D265A) attached with histidine-tag at the C-terminus. Retroviral infection of NIH3T3, 38B9, CT26 or MC/9 cells were previously described^7^.

### Establishment of mAbs specific to iSEC1

SD rats were treated twice with footpad injection of iSEC1-expressing NIH3T3 transfectants (5 × 10^7^ cells each), first with CFA (Sigma-Aldrich), and two weeks later with IFA (Sigma-Aldrich). Three days after the second immunization, cells isolated from popliteal lymph nodes were fused with X63.Ag8-653 cells using polyethylene glycol 1500 (Roche Diagnostics). B cell hybridomas were selected in medium containing hypoxanthine/aminopterin/thymidine (GIBCO/Invitrogen) and recombinant IL-6, and their culture supernatants were screened for the ability to react with iSEC1- or iSEC2-expressing NIH3T3 transfectants but not untransfected NIH3T3 cells.

### RT-PCR analysis

Total RNA was prepared from tissues or cells, and subjected to first-strand cDNA synthesis with reverse transcription using oligo dT primers. PCR was performed with cDNA templates using following primers: forward for *Isec1*, 5′-ctttcagttcagctaggaaacac-3′, for *Isec2*, 5′-gtaggaggaaattacattgtccg-3′, and reverse for both *Isec1* and *Isec2*, 5′-cttaccttttcttttggaaataaat-3′, and for *Cd200*, forward 5′-ttactgcggcccagagcaaggatg-3′, and reverse 5′-gctcttatttcattctttgcatcccctg-3′ and for *Actb*, forward 5′-gatgacgatatcgctgcgctg-3′ and reverse 5′-gtacgaccagaggcatacagg-3′.

Quantitative-PCR was performed with following primers, *Il2*: forward 5′-aactccccaggatgctcac-3′, and reverse 5′-cgcagaggtccaagttcatc-3′, *Ifng*: forward 5′-ggatggtgacatgaaaatcctgc-3′, and reverse 5′-tgctgatggcctgattgtctt-3′, *Tnf*: forward 5′-gcctcttctcattcctgcttg-3′, and reverse 5′-gggtctgggccatagaactg-3′, *Hprt*: forward 5′-ggccagactttgttggatttg-3′, and reverse 5′-cgctcatcttaggctttgtatttg-3′, *Krt19*: forward 5′-caaaacctcaatgatcgtctcgc -3′, and reverse 5′-gcgcaccttgtccaagtag-3′, *Lct*: forward 5′-caggggaatgactgggaatct-3′, and reverse 5′-gcccgaagactgctgaagt-3′, *Muc2*: forward 5′-caagggctcggaactccag-3′, and reverse 5′-ccagggaatcggtagacatcg-3′, *Chga*: forward 5′-ccaaggtgatgaagtgcgtc-3′, and reverse 5′-ggtgtcgcaggatagagagga-3′, *Lyz1*: forward 5′-gagaccgaagcaccgactatg-3′, and reverse 5′-cggttttgacattgtgttcgc -3′.

### Cell surface and intracellular staining

After pre-incubation with anti-CD16/32 (2.4G2) and normal rat serum to prevent non-specific binding of irrelevant Abs, cells were stained with indicated combination of Abs, and analyzed with a FACSCanto II (BD Biosciences). Binding of IgFc fusion proteins to the cell surface was detected by staining with APC-conjugated anti-mouse IgG1 mAb. For intracellular staining, cytospin preparations were fixed with 4% paraformaldehyde, pre-treated with blocking solution containing 1% BSA, and incubated with indicated rabbit-derived antibodies, followed by FITC-conjugated goat anti-rabbit IgG. Stained cells were examined under fluorescence microscope (Olympus). For the whole mount staining of the intestine, mice were treated with intraperitoneal administration of 30 μg of Alexa Fluor647 (Life Technologies, A20173)-conjugated anti-iSEC1 (GT42) or isotype-matched control antibody. Sixteen hrs later, the small intestine was isolated and examined under confocal fluorescence microscope (A1, Nikon).

### Immunoblotting

Cells were treated with lysis buffer containing 1% Nonidet P-40 (Sigma-Aldrich) and protease inhibitor cocktail (Roche Diagnostics), and cell lysates were subjected to SDS-PAGE. FLAG-tagged proteins were detected with anti-FLAG-HRP conjugate, and visualized by Immobilon Western (Millipore).

### Measurement of cytokine production

IL-2-cultured IELs were starved of IL-2 for 3 h, and then stimulated for 24 h in 96-well plates that had been coated with anti-CD3 mAb (0.2 μg/ml) plus His-tagged proteins (5 μg/ml of iSEC1-IgFc, CD200-IgFc or control FLAG-IgFc) captured via anti-histidine antibody (5 μg/ml). Cells were subjected to RT-PCR analysis for cytokine mRNA expression, and secreted cytokines (IL-2, IFNγ and TNFα) were measured by using CBA Enhanced Sensitivity Flex Set (BD Bioscience).

### Cytolysis assay

CT26 transfectants (1 × 10^4^ as target cells) expressing GFP together with iSEC1, CD200, CD200R or mock were seeded in the 96 well flat-bottom plates and incubated for 6 h to let them adhere to the plate. IL-2-cultured IELs were added at indicated E/T ratio on CT26 transfectants, and cultured for 48 h in 100 μl/well of complete IMDM supplemented with 40 ng/ml IL-2. Dead and floating cells were then removed by washing wells, and the GFP fluorescence intensity of remaining CT26 transfectants was measured by using GloMax-Multi Detection System (Promega Corporation). % lysis = (1 - GFP intensity when co-cultured with T cells/GFP intensity when cultured without T cells) × 100.

### Statistical analysis

Statistical analysis was performed with unpaired Student’s t test. A p value < 0.05 was considered statistically significant.

## Additional Information

**How to cite this article**: Kojima, T. *et al.* Novel CD200 homologues iSEC1 and iSEC2 are gastrointestinal secretory cell-specific ligands of inhibitory receptor CD200R. *Sci. Rep.* **6**, 36457; doi: 10.1038/srep36457 (2016).

**Publisher’s note**: Springer Nature remains neutral with regard to jurisdictional claims in published maps and institutional affiliations.

## Supplementary Material

Supplementary Information
