# Supplementary material for: Novel CD200 homologues iSEC1 and iSEC2 are gastrointestinal secretory cell-specific ligands of inhibitory receptor CD200R
Source: Sci Rep. 2016 Nov 7;6:36457. doi: 10.1038/srep36457 (PMC5098219; doi:10.1038/srep36457)
Supplement: Supplementary Information [file srep36457-s1.doc]

**Novel CD200 homologues iSEC1 and iSEC2 are gastrointestinal secretory cell-specific ligands of inhibitory receptor CD200R**

Toshiyuki Kojima^1^, Kiichiro Tsuchiya^2^, Shinji Ikemizu^3^, Soichiro Yoshikawa^1^, Yoshinori Yamanishi^1^, Mamoru Watanabe^2^, and Hajime Karasuyama^1,*^

^1^Department of Immune Regulation, ^2^Department of Gastroenterology and Hepatology, Tokyo Medical and Dental University, Graduate School of Medical and Dental Sciences. Tokyo 113-8519, Japan. ^3^Division of Structural Biology, Graduate School of Pharmaceutical Sciences, Kumamoto University. Kumamoto 862-0973, Japan

*Correspondence: Hajime Karasuyama


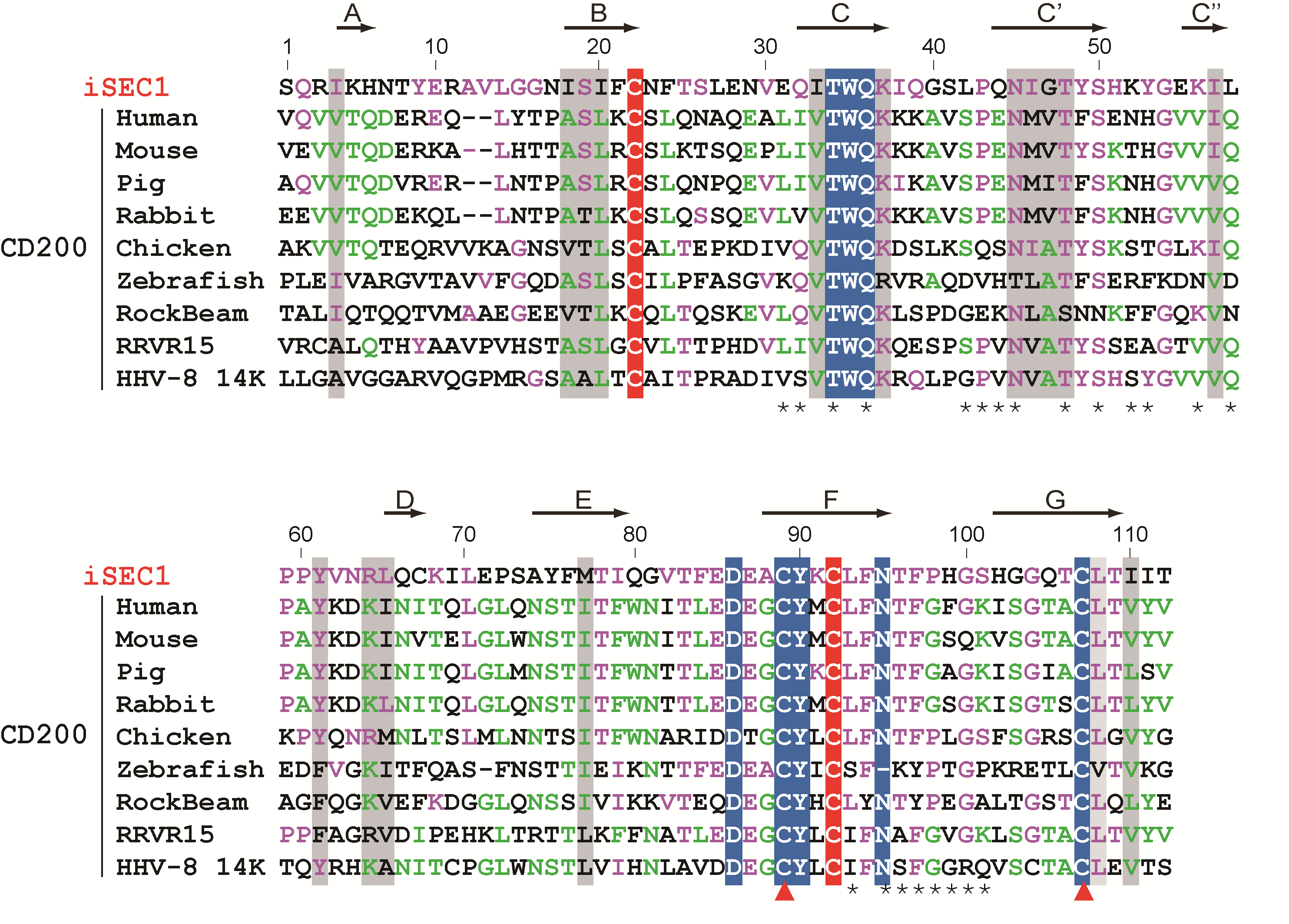


**Supplementary Figure 1: iSEC1 displays the similarity to CD200 in the IgV-like domain.**

Amino acid sequences of the IgV-like domain of CD200 derived from seven different animal species and two CD200-like proteins derived from virus (RRVR15 and HHV-8) are aligned with that of iSEC1 on the same criteria described in Fig. 1. Residues shared by all 10 proteins are highlighted in blue while similar residues are shaded. Conserved cysteine residues for IgSF-domain formation are highlighted in red. Residues identical more than five proteins are indicated by green letters. Residues identical between iSEC1 and any other proteins are indicated by magenta letters. Asterisks denote residues at the CD200/CD200R interface^12^. Extra cysteine residues conserved in CD200 orthologues are indicated by red arrowheads. Amino acid sequences are retrieved from the NCBI data base: human (NP_005935), mouse (NP_034948), pig (XP_003358857), rabbit (NP_001316003), chicken (NP_001025958), zebrafish (XP_017211448), rock bream (BAM36394), RRVR15 (AAD21407) and HHV-8 14k (AAK53417).

**
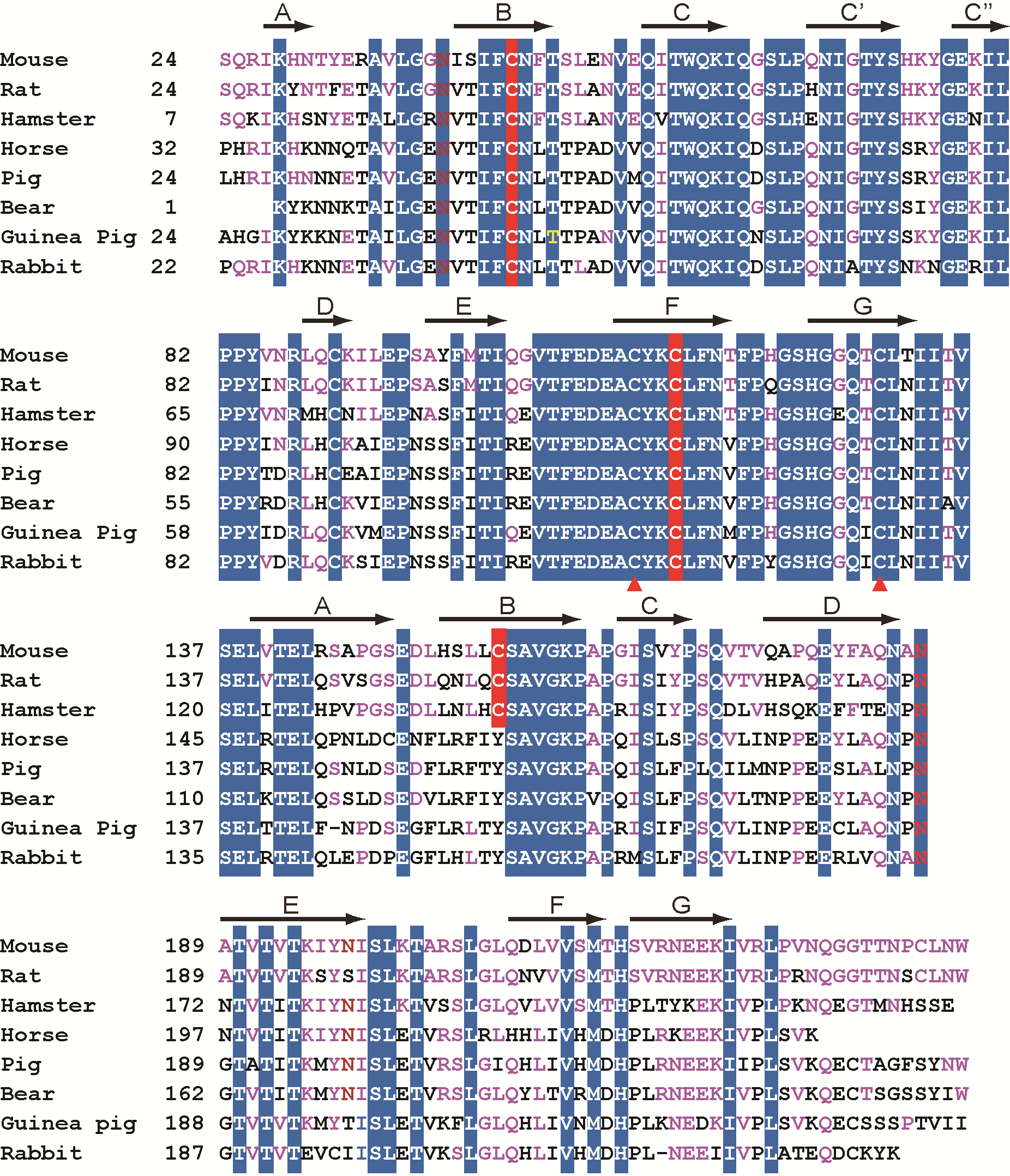
**

**Supplementary Figure 2: Amino acid alignments of iSEC1 orthologues.**

Amino acid sequences of the IgV- and IgC-like domains of iSEC1 orthologues derived from 8 different animal species are aligned on the same criteria described in Fig. 1. Residues shared by all 8 proteins are highlighted in blue. Conserved cysteine residues for IgSF-domain formation are highlighted in red. Residues identical between mouse iSEC1 and any other proteins are indicated by magenta letters. Potential N-glycosylation sites are denoted by red letters. Extra cysteine residues conserved in CD200 orthologues are indicated by red arrowheads. Amino acid sequences are retrieved from the NCBI database: rat (XP_003752537.2), hamster (XP_005074863.1), pig (XP_003483369.1), rabbit (XP_004578354.1), horse (XP_008528716.1), bear (XP_008704816.1), guinea pig (XP_013013580.1).

**
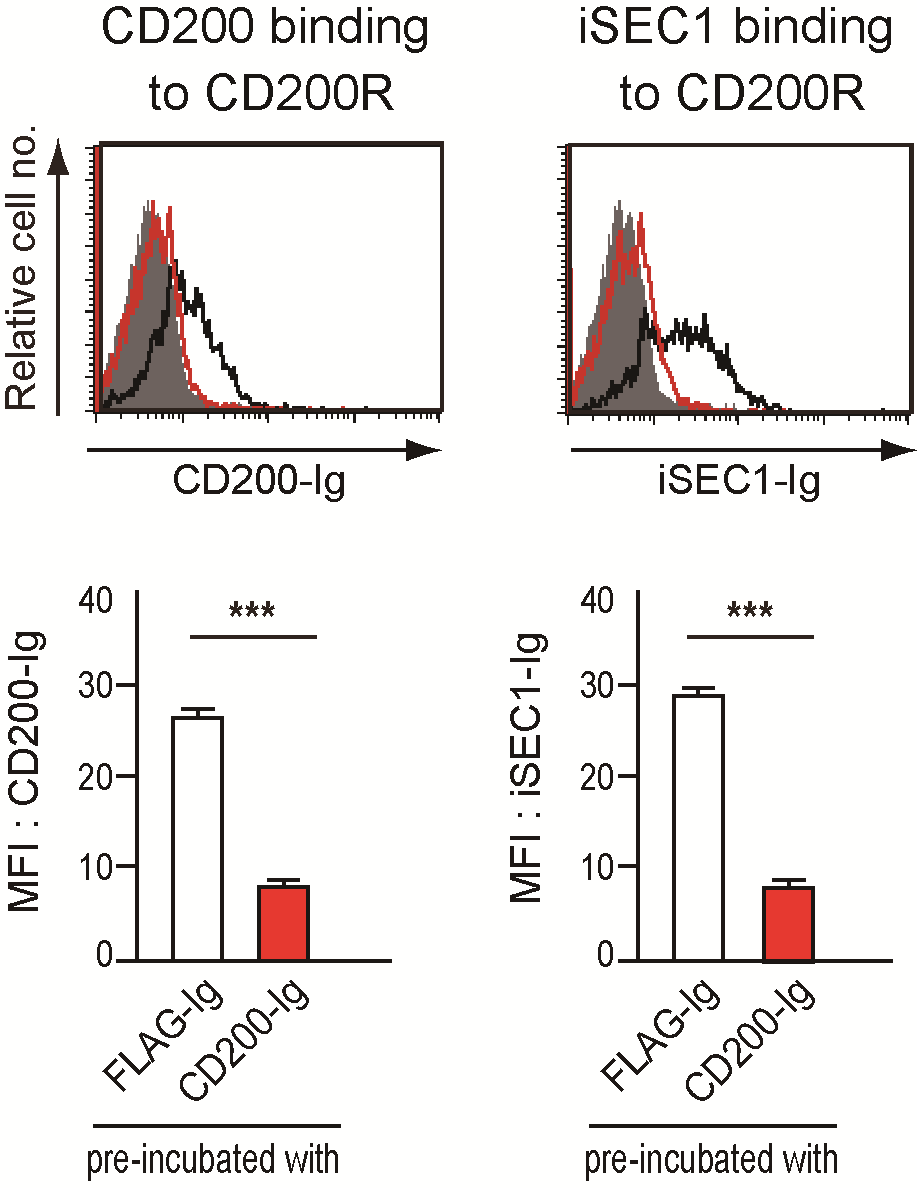
**

**Supplementary Figure 3:** **The iSEC1-CD200R1 interaction is inhibited by CD200.**

CD200R-expressing NIH3T3 transfectants were first pre-incubated with FLAG-IgFc (black histograms in upper panels), CD200-IgFc (red histograms in upper panels), and then incubated with His-tagged CD200-IgFc (left panel) or iSEC1-IgFc (right panel). Binding of His-tagged proteins to CD200R expressed on the transfectants was detected by fluorescent-labeled anti-His antibody. Shaded histograms show control staining with His-tagged FLAG-IgFc. All the data are summarized in lower panels, in that the mean fluoresce intensity of CD200-IgFc or iSEC1-IgFc staining in each group is shown (mean ± SEM, n=3). Data shown are representative of three independent experiments. p***<0.001.


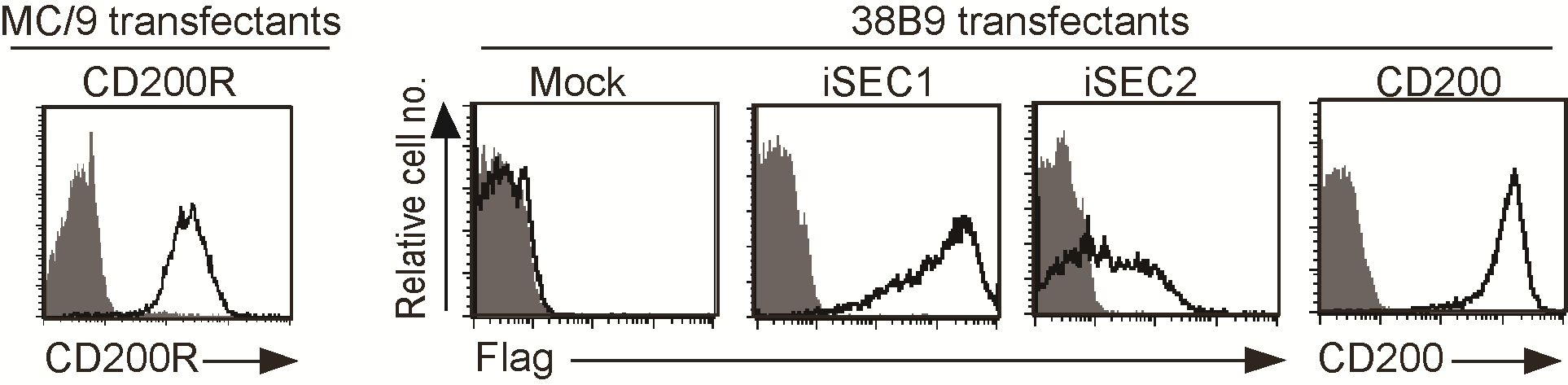


**Supplementary Figure 4: Establishment of transfectants expressing CD200R or its ligands.** MC/9 cells were infected with retroviral vectors encoding RFP plus CD200R while 38B9 cells were infected with retroviral vectors encoding GFP together with mock control, FLAG-tagged iSEC1 or iSEC2, or CD200. Cell surface expression of indicated proteins (open histograms) was detected by staining with anti-CD200R (OX110), anti-FLAG, or anti-CD200 (OX90) mAb. Shaded histograms indicate staining with isotype-matched control Ab. Data shown are representative of three independent experiments.


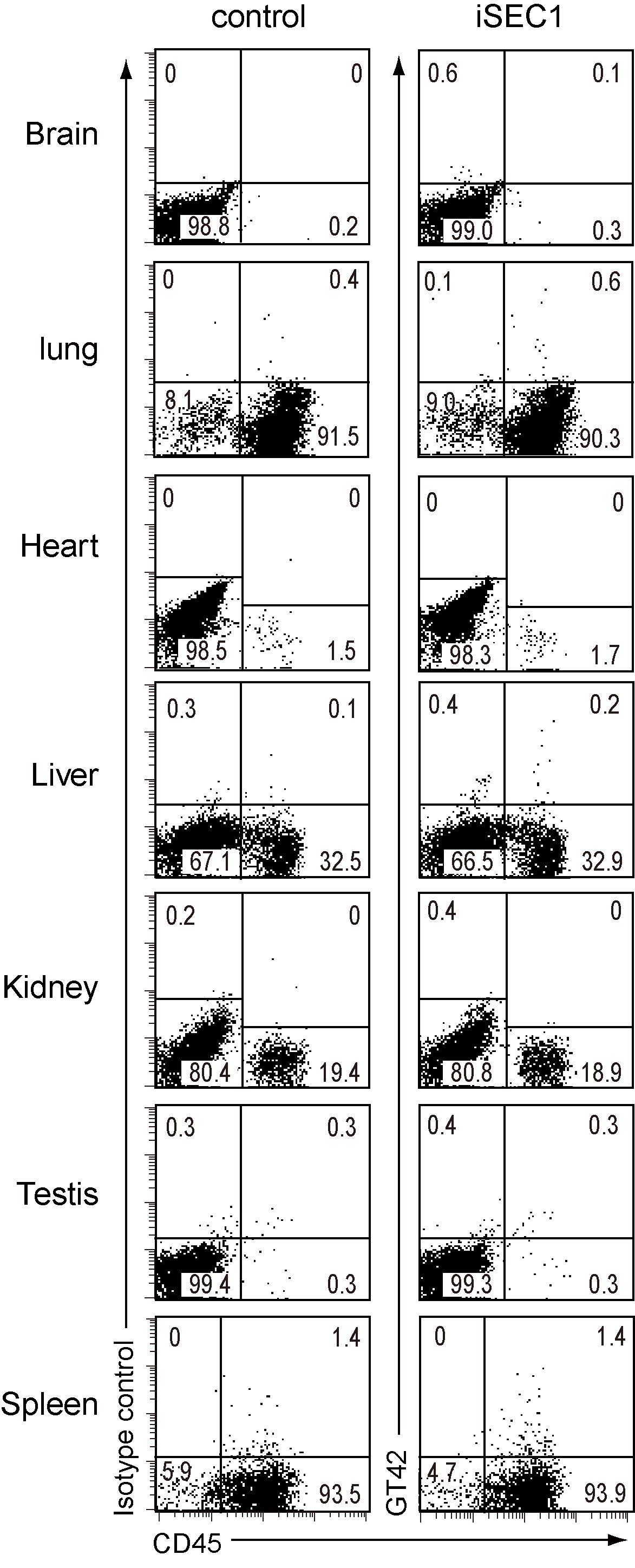


**Supplementary Figure 5: No detectable expression of iSEC1 in non-gastrointestinal tissues.** Cells were isolated from indicated organs and stained with anti-CD45 in combination with anti-iSEC1 or isotype-matched control. Data shown are representative of three independent experiments.


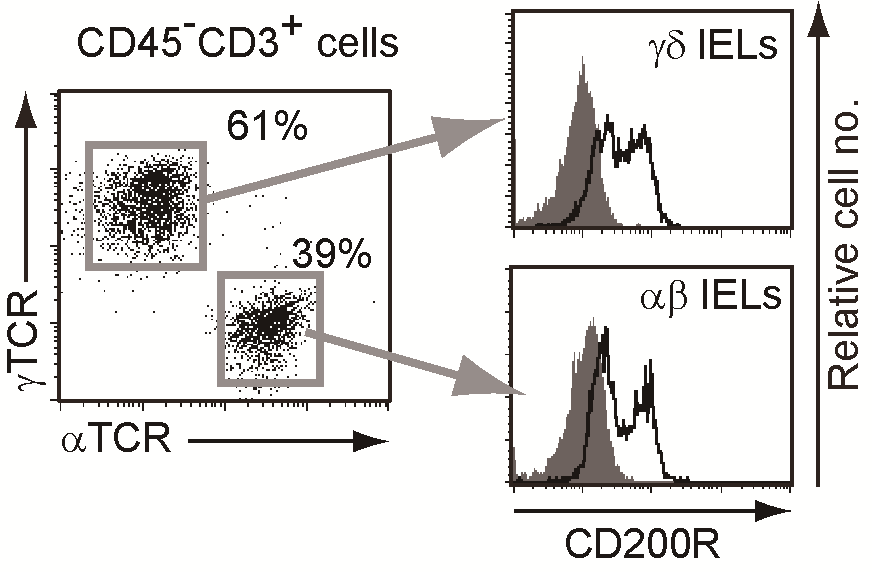


**Supplementary Figure 6: Both T cell receptor αβ- and γδ-IELs express CD200R.**

Left panel shows the expression of α T cell receptor (TCR) and γ TCR on CD45^+^CD3^+^ T cells isolated from the jejunum as in Fig. 6. Right panels show the CD200R expression on αβ- and γδ- T cells. Shaded histograms show control staining with isotype-matched antibody. Data shown are representative of three independent experiments.


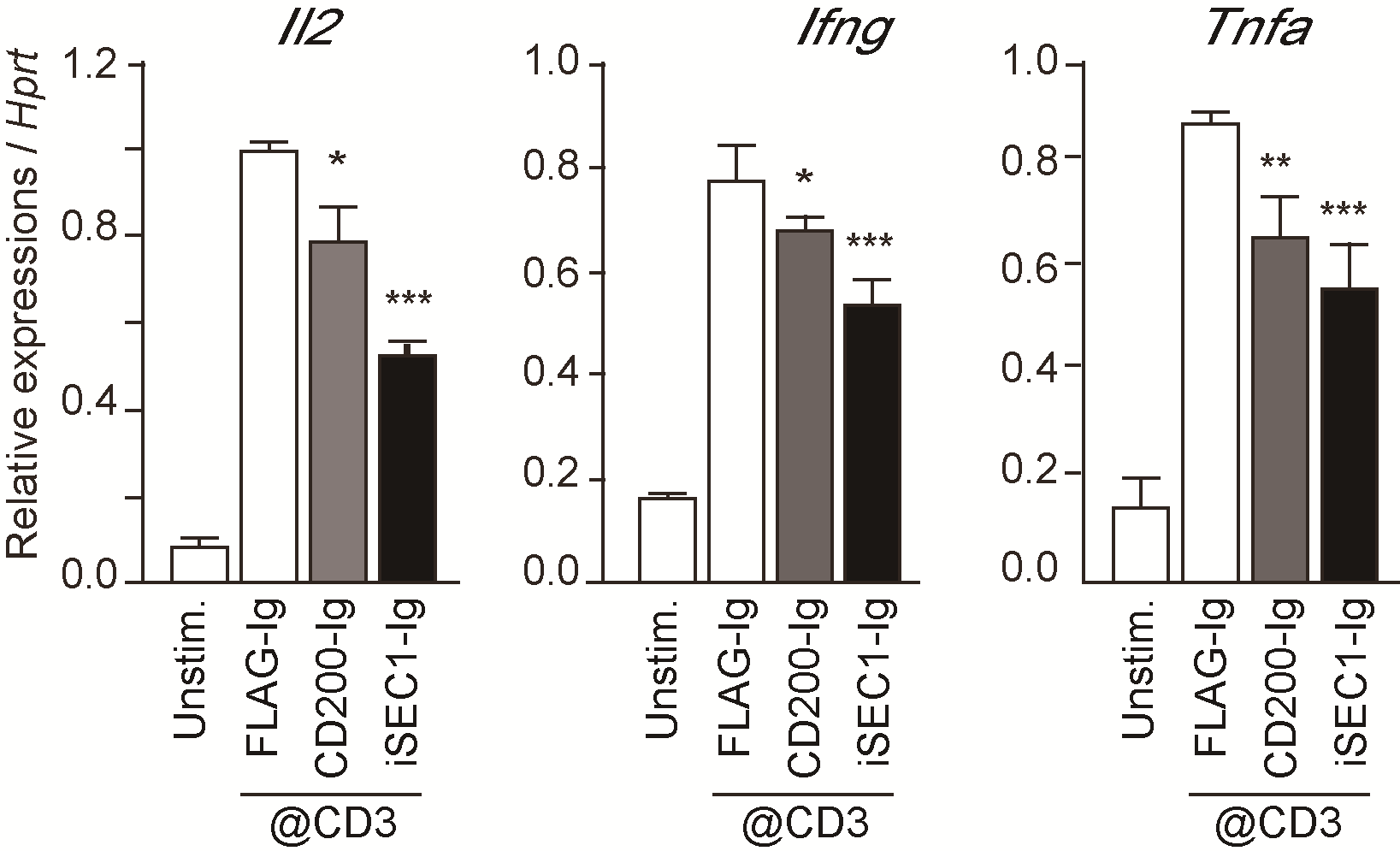


**Supplementary Figure 7: Binding of iSEC1 to CD200R on IELs attenuates their cytokine expression.** As shown in Fig. 6c, IL-2-cultured IELs were left unstimulated or stimulated for 24 h with plate-bound anti-CD3 in the presence of plate-bound mock IgFc, CD200-IgFc or iSEC1-IgFc. RNAs were prepared and subjected to quantitative RT-PCR analysis for the expressions of indicated cytokine genes (mean ± SEM, n=3). Data shown are representative of three independent experiments.
